# Evaluation of AI diagnostic systems for breast ultrasound: comparative analysis with radiologists and the effect of AI assistance

**DOI:** 10.1007/s11604-025-01809-2

**Published:** 2025-06-09

**Authors:** Sayumi Tsuyuzaki, Tomoyuki Fujioka, Emi Yamaga, Leona Katsuta, Mio Mori, Yuka Yashima, Mayumi Hara, Arisa Sato, Iichiroh Onishi, Jitsuro Tsukada, Tomoyuki Aruga, Kazunori Kubota, Ukihide Tateishi

**Affiliations:** 1 Department of Diagnostic Radiology, Institute of Science Tokyo, 1-5-45 Yushima, Bunkyo-ku, Tokyo 113-8519 Japan; 2 Department of Radiology, Dokkyo Medical University Saitama Medical Center, 2-1-50 Minamikoshigaya, Koshigaya, Saitama 343-8555 Japan; 3 Department of Radiology, Kanto Central Hospital of the Mutual Aid Association of Public School Teachers, 6-25-1 Kamiyoga, Setagaya-ku, Tokyo 158-8531 Japan; 4 Nitobe-Memorial Nakano General Hospital, 4-59-16 Tyuo, Nakano-ku, Tokyo 164-8607 Japan; 5 Department of Diagnostic Pathology, Institute of Science Tokyo, Bunkyo-ku, Tokyo 113-8510 Japan; 6 Department of Diagnostic Radiology, Keio University School of Medicine, 35, Shinanomachi, Shinjyuku-ku, Tokyo 160-8582 Japan; 7 Department of Breast Surgery, Institute of Science Tokyo, Bunkyo-ku, Tokyo 113-8510 Japan

**Keywords:** Breast ultrasound, Artificial intelligence (AI), Computer-aided diagnosis (CADx), BI-RADS, Diagnostic accuracy, Radiologist efficiency

## Abstract

**Purpose:**

The purpose of this study is to evaluate the diagnostic accuracy of an artificial intelligence (AI)-based Computer-Aided Diagnosis (CADx) system for breast ultrasound, compare its performance with radiologists, and assess the effect of AI-assisted diagnosis. This study aims to investigate the system’s ability to differentiate between benign and malignant breast masses among Japanese patients.

**Materials and Methods:**

This retrospective study included 171 breast mass ultrasound images (92 benign, 79 malignant). The AI system, BU-CAD™, provided Breast Imaging Reporting and Data System (BI-RADS) categorization, which was compared with the performance of three radiologists. Diagnostic accuracy, sensitivity, specificity, and area under the curve (AUC) were analyzed. Radiologists’ diagnostic performance with and without AI assistance was also compared, and their reading time was measured using a stopwatch.

**Results:**

The AI system demonstrated a sensitivity of 91.1%, specificity of 92.4%, and an AUC of 0.948. It showed comparable diagnostic performance to Radiologist 1, with 10 years of experience in breast imaging (0.948 vs. 0.950; *p* = 0.893), and superior performance to Radiologist 2 (7 years of experience, 0.948 vs. 0.881;* p* = 0.015) and Radiologist 3 (3 years of experience, 0.948 vs. 0.832;* p* = 0.001). When comparing diagnostic performance with and without AI, the use of AI significantly improved the AUC for Radiologists 2 and 3 (*p* = 0.001 and 0.005, respectively). However, there was no significant difference for Radiologist 1 (*p* = 0.139). In terms of diagnosis time, the use of AI reduced the reading time for all radiologists. Although there was no significant difference in diagnostic performance between AI and Radiologist 1, the use of AI substantially decreased the diagnosis time for Radiologist 1 as well.

**Conclusion:**

The AI system significantly improved diagnostic efficiency and accuracy, particularly for junior radiologists, highlighting its potential clinical utility in breast ultrasound diagnostics.

## Introduction

Breast cancer remains a significant global health challenge and is the most diagnosed cancer in women worldwide, making it the leading cause of cancer death among women[1]. According to the latest cancer statistics in the United States, breast cancer accounts for 32% of all new cancer diagnoses among women. Female breast cancer incidence rates have been slowly increasing by about 0.6% per year since the mid‐2000 s [1]. However, despite the increase in incidence, female breast cancer mortality peaked in 1989 and has since decreased by 42% through 2021. This is attributed to effective screening programs and improved treatment [1].

One breast cancer screening modality is ultrasonography, which we widely use because it is minimally invasive and low-cost [2, 3]. We know that mammography is the only proven method that reduces mortality in breast cancer screening. However, it is not particularly suitable for younger women or those with dense breast tissue. Moreover, Asian populations, including Japanese women, have a higher prevalence of dense breast tissue, making mammography less effective in these groups. Therefore, incorporating ultrasound is essential to improve breast cancer screening efficacy in such populations [4–6]. In response, a study called the Japan Strategic Anti-Cancer Randomized Trial (J-START) was conducted to investigate the efficacy of adjunctive ultrasonography in Japanese patients [7]. In this study, women were divided into two groups: one group underwent both mammography and ultrasonography, while the other group underwent mammography alone. This study demonstrated that adjunctive ultrasonography increases sensitivity and the detection rate of early cancers. As previous studies have shown, it is important to effectively use breast ultrasonography in cancer screening and routine clinical practice. However, ultrasonography has the disadvantage of poor reproducibility and operator dependency, which needs to be addressed [8].

To address these challenges, artificial intelligence (AI) has emerged as a promising solution, enhancing diagnostic accuracy across various medical imaging applications, including image classification, lesion detection, segmentation, image quality improvement, and prognostic prediction. In the domain of breast imaging, the utility of AI has been demonstrated across multiple modalities, including mammography, ultrasound, Magnetic Resonance Imaging, and Positron Emission Tomography/Computed Tomography [2, 9–14].

Most studies evaluating the applicability of AI in breast imaging diagnostics have focused on Western populations, with limited research on Asians, including Japanese [15–17]. Given the high prevalence of dense breast tissue among Japanese women, there is a need for independent AI research to demonstrate the usefulness of breast ultrasound AI for Japanese women. In this study, we evaluated the accuracy of a Computer-Aided Diagnosis (CADx) system for breast ultrasound developed in Taiwan in differentiating between benign and malignant breast masses in Japanese patients and assessed its potential for clinical application [18–20]. Furthermore, we compared the diagnostic accuracy of the CADx system to that of radiologists, examining whether the use of AI improves diagnostic accuracy and enables more efficient image reading.

## Materials and methods

### Patients

Our medical ethics committee approved this retrospective study and waived the requirement for written informed consent from patients (approval number: M2019-232; approval date: 2019.12.13). All methods were carried out in accordance with relevant guidelines and regulations (Declaration of Helsinki).

The subjects were patients who underwent breast ultrasound examinations at Tokyo Medical and Dental University Hospital from July 2021 to May 2022. The inclusion criteria were female patients whose masses were diagnosed as benign or malignant by pathological analysis or by > 1 year follow-up examinations at our hospital. The exclusion criteria were (a) patients who were treated with hormonal therapy, chemotherapy, or radiation therapy to avoid potential confounding effects on tumor characteristics, (b) patients who were < 20 years old, (c) patients diagnosed with recurrent breast cancer, and (d) patients who were unable to express their consent due to illness or advanced age.

A breast radiologist and a medical student reviewed the database of radiology reports and clinical records at our institute.

The patients underwent the examination in a supine position with their arms up. The ultrasound examinations, image acquisition, and biopsies were performed by breast radiologists. The radiologists acquired static images in the vertical and horizontal planes and measured the maximum diameter of the masses. The US systems we used were an Aplio 500 scanner with an 8.0 MHz linear PLT-805 AT (Canon Medical Systems Corporation, Tochigi, Japan), or a LOGIC E10 s scanner with a linear matrix ML6-15-D (GE Healthcare, Chicago, IL, USA).

### Data set

Table 1 shows the characteristics of patients and masses. A total of 171 static images of each breast mass (92 benign, 79 malignant) extracted from ultrasound examinations. A single patient could have up to four lesions included in the dataset, with a maximum of two lesions per breast per patient. In cases where a patient had multiple lesions, a breast radiologist who was not one of the three radiologists described in the Image Evaluation and Statistical Analysis section selected the most representative lesions. Priority was given to lesions with higher Breast Imaging Reporting and Data System (BI-RADS) categories or those with better image quality [21]. All solid and cystic masses were evaluated in this study. Table 2 shows the histopathological results of the masses. Among the malignant cases, six were ductal carcinoma in situ, while the rest were invasive carcinoma.

**Table 1:** Characteristics of patients and masses

	Benign	Malignant	All	*p* value benign versus malignant
Masses (n)	92	79	171	
Age				
Mean (y)	52.3 ± 14.6	61.4 ± 13.1	56.5 ± 14.6	< 0.001
Range (y)	22.5–85.1	37.9–89.6		
Maximum diameter				
Mean (mm)	10.8 ± 7.0	18.2 ± 7.8		< 0.001
Range (mm)	4–41	7–39		
Minimum diameter				
Mean (mm)	8.6 ± 6.4	14.3 ± 6.6		< 0.001
Range (mm)	2–36	5–37

**Table 2:** Histopathology of masses

Benign (*n* = 92)	Malignant (*n* = 79)
Fibroadenoma 7	Ductal carcinoma in situ 6
Intraductal papilloma 1	Invasive ductal carcinoma 65
Mastopathy 2	Apocrine carcinoma 5
Hamartoma 1	Invasive lobular carcinoma 2
Cyst 2	Invasive secretory carcinoma 1
Ductal hyperplasia 1	
Intramammary lymph nodes 1	
Not known 77 (diagnosed by follow-up)	

### AI diagnostic support system

**Fig.1:**
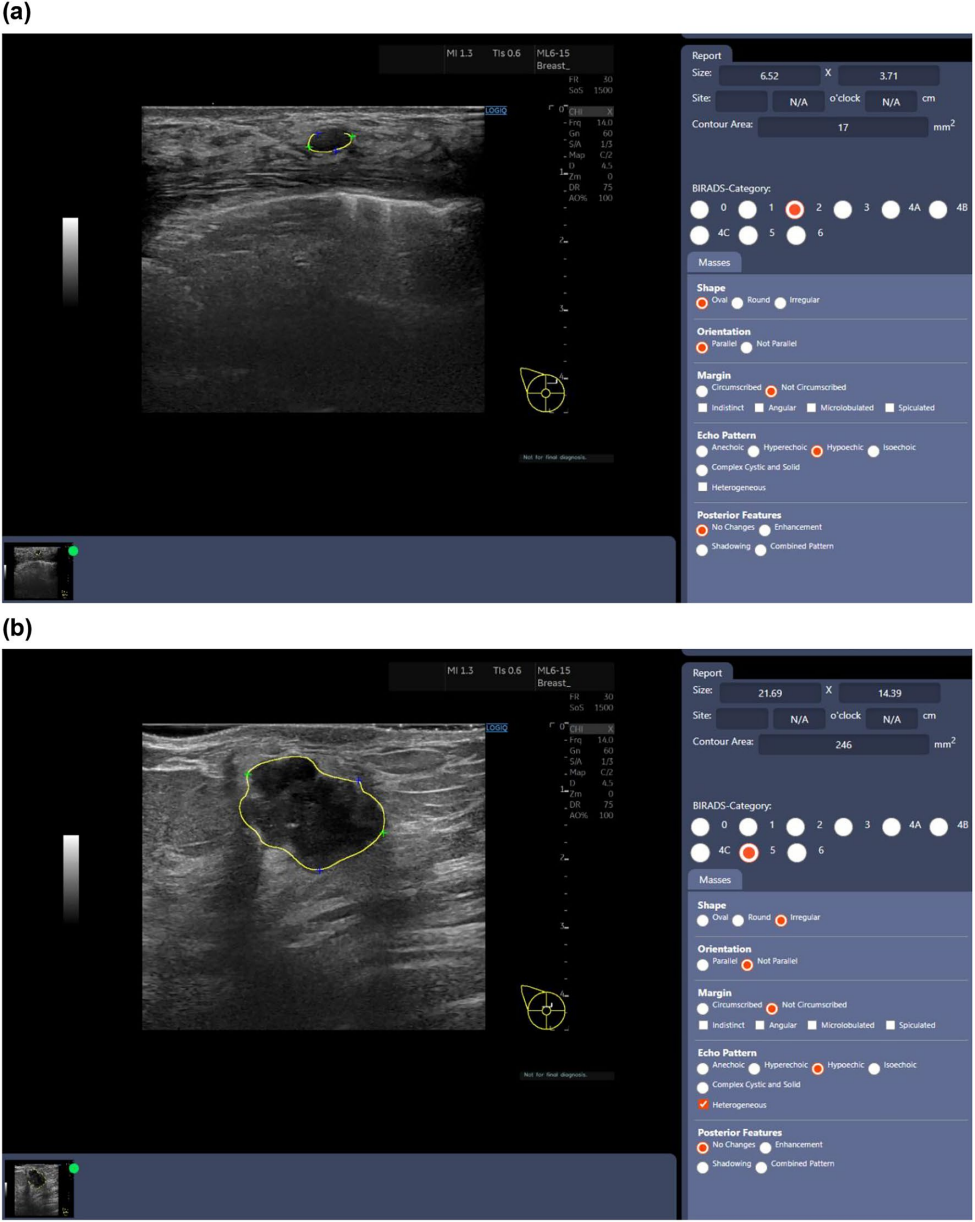
Two examples of the Results of the BU-CAD™ System. **a** A benign case (fibroadenoma) of a 70-year-old patient. BI-RADS Category: 2. **b** A malignant case (invasive ductal carcinoma) of a 60-year-old patient. BI-RADS Category: 5

The images were analyzed using the AI diagnostic support system (BU-CAD^™^, TaiHao Medical Inc., Taipei City, Taiwan), which is approved in Taiwan, and the United States and provides Breast Imaging Reporting (findings, category and assessment) based on BI-RADS [21]. When images are input into BU-CAD™, the system performs segmentation of any identified lesions and indicates their sizes. It then provides information on various attributes, including BI-RADS categories (from 1 to 5), shape (oval, irregular, etc.), orientation (parallel, not parallel), margin (circumscribed or not), echo pattern (hyperechoic, hypoechoic, etc.), and posterior features (enhancement, shadowing, etc.). Figure 1 shows the examples of the successful diagnoses of the AI system. The upper one is that of benign, whose BI-RADS category is 2, and the lower is that of malignant, whose BI-RADS category is 5.

The effectiveness of this AI system has already been evaluated and demonstrated in a study with patients in Taiwan [22]. The BU-CAD™ was trained by using data consisted of 1225 breast ultrasound patient cases (mean age: 45.58 ± 9.62 years; range: 17–85 years) with 1687 biopsy-proven lesions (mean diameter: 14.51 ± 8.03 mm; range 2.3–45 mm) collected from one institution in Taiwan. Table 3 shows lesion type distributions of training dataset. Among them, 126 cases are challenging cases which BI-RADS category from radiology report are less than or equal to 3 but the biopsy results are malignant. In addition, breast ultrasound images without any lesion from the 1225 cases were used as negative images for the training process of Soft Tissue Lesion Identification Module.

**Table 3:** Training data in the BU-CAD^™^ system

Benign type	No. of lesions	Malignant type	No. of lesions
FA	503	IDC	663
FC	404	DCIS	40
Papilloma	17	ITC	12
Others	29	Others	19
Total no. of lesions	953	Total no. of lesions	734

*FA* Fibroadenoma, *FC* Fibrocystic Changes, *IDC* Invasive Ductal Carcinoma, *DCIS* Ductal Carcinoma In Situ, *ITC* Isolated Tumour Cells. A very small cluster of cancer cells identified in lymph nodes or other tissues., *no.* Number

### Image evaluation and statistical analysis

The assessed BI-RADS categories were reported as 2, 3, 4a, 4b, 4c, or 5 by the readers [21]. First, the images were analyzed using the AI system alone. Subsequently, three breast radiologists (Radiologist 1 with 10 years, Radiologist 2 with 7 years, and Radiologist 3 with 3 years of experience in breast imaging) independently categorized these images using BI-RADS without the AI system. More than one month later, the radiologists re-categorized these images using BI-RADS with the AI system. In both cases, reading time for each radiologist was recorded and analyzed. The reading time was measured as the duration from the moment the ultrasound image was displayed on the screen until the reader completed the assessment by assigning a BI-RADS category and recording the percentage likelihood of malignancy. For each case, the readers self-reported the reading time using a stopwatch. The standalone diagnostic accuracy of the AI system was also evaluated.

In this research, BI-RADS categories 4 A or higher were regarded as malignant to calculate the sensitivities and specificities. The BI-RADS lexicon recommends a biopsy when a malignancy probability is 2% or higher (BI-RADS category 4 and above), so the cut-off value for AI and radiologists was set at 2% of likelihood of malignancy. The diagnostic performances (sensitivity, specificity, and area under the curve (AUC)) of the radiologist and the AI system were calculated and compared in terms of accuracy. using SPSS for Windows version 29.0 (IBM Corp., New York, USA). Receiver Operating Characteristic (ROC) curve comparisons were conducted using DeLong’s test, and paired t-tests were used to compare diagnostic times. We considered *p* value of < 0.05 as statistically significant.

## Results

Table 1 shows the characteristics of masses. There were significant differences in size (maximum diameter, minimum diameter) between benign and malignant lesions (*p* < 0.001). Of the 92 benign lesions, 77 were diagnosed by follow-up.

**Fig.2:**
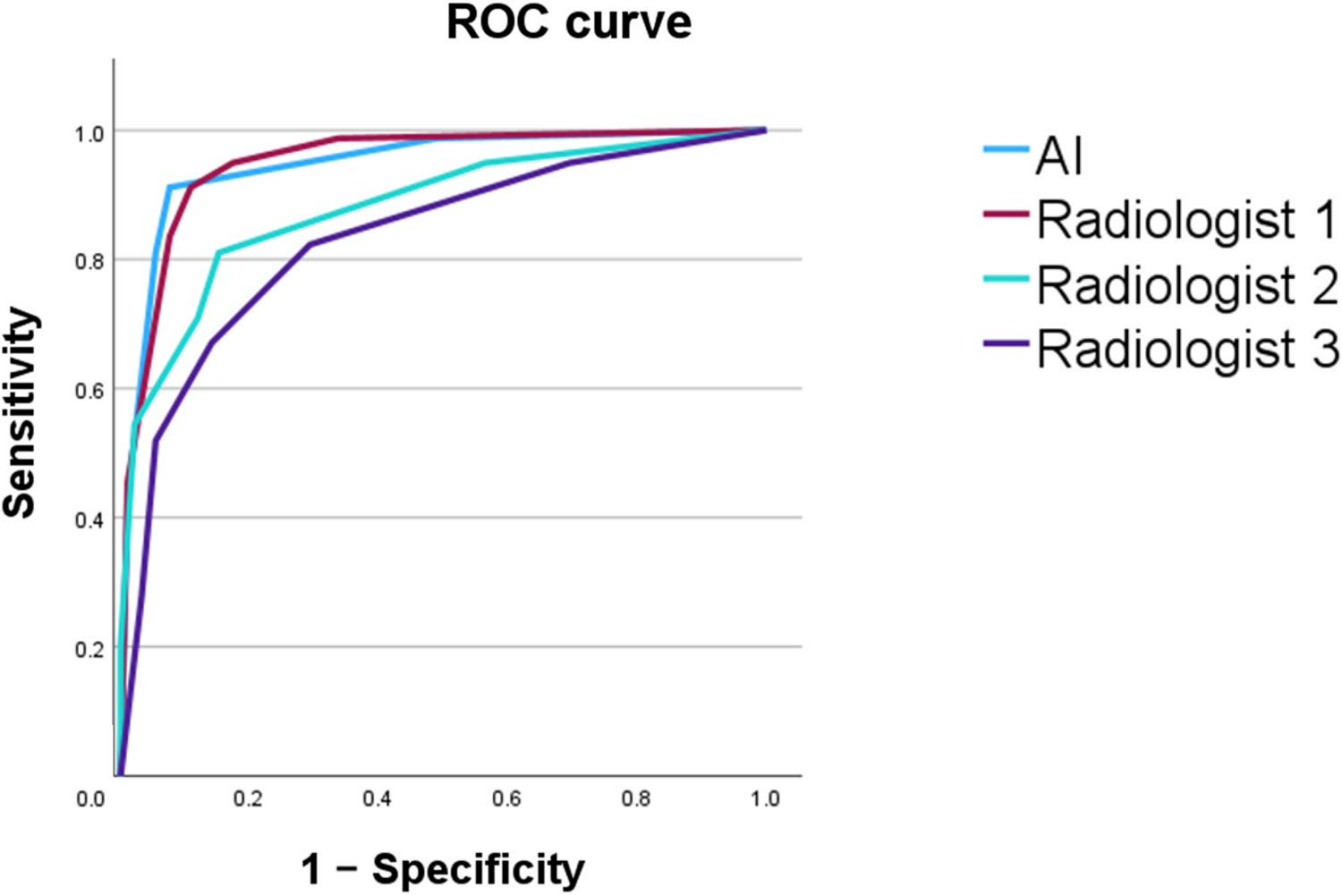
ROC curves of both AI and radiologists’ performance

Table 4 shows diagnostic performance and Fig. 2 demonstrates the ROC curves of both AI and radiologists’ performance. The *p* values of AUC were also calculated between AI and each radiologist. As shown in Fig. 2, the AI system achieved an AUC comparable to Radiologist 1, while demonstrating superior diagnostic performance compared to Radiologist 2 and Radiologist 3.

**Table 4:** Diagnostic performance of both AI and radiologists

	Sensitivity	Specificity	AUC	*p* value (vs AI)
AI	0.911	0.924	0.948	
Radiologist1	0.949	0.826	0.950	0.893
Radiologist2	0.810	0.848	0.881	0.015
Radiologist3	0.823	0.707	0.832	0.001

*AI* artificial intelligence, *AUC* Area Under the Receiver Operating Characteristic Curve

AI showed a sensitivity of 91.1%, a specificity of 92.4%, and an AUC of 0.948. AI showed comparable AUC to Radiologist 1, 10 year experience in breast imaging (0.948 vs. 0.950; *p* = 0.893), and showed a significantly higher AUCs than Radiologist 2, 7 year experience in breast imaging (0.948 vs. 0.881; *p* = 0.015) and Radiologist 3, 3 year experience in breast imaging (0.948 vs. 0.832; *p* = 0.001).

**Fig.3:**
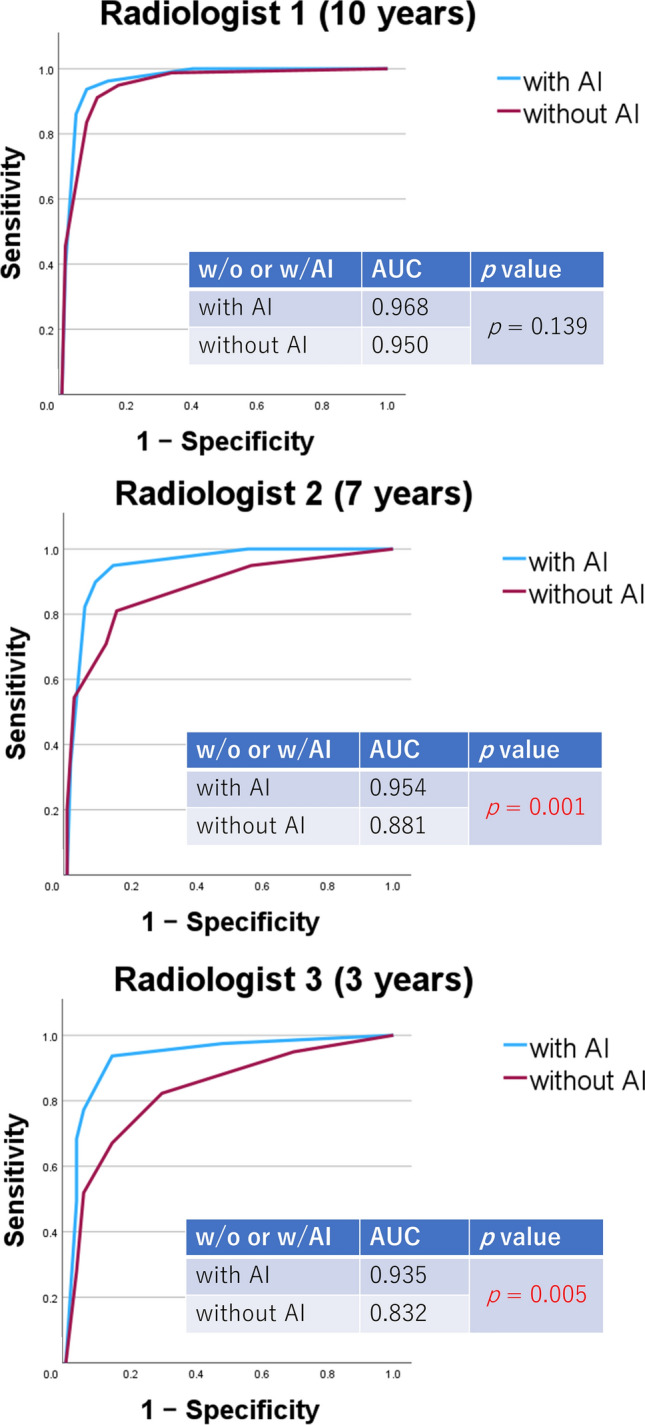
ROC curves and AUC of each radiologist with or without AI. “w/o” indicates without AI, and “w/AI” indicates with AI

Figure 3 shows the ROC curves and AUC of each radiologist with or without AI. Blue lines are with AI and violet lines are without AI. The *p* values of AUC on the same radiologist were also calculated. The AUCs of Radiologists 2 and 3 with AI were significantly larger than those without AI (*p* = 0.001 and 0.005 for each Radiologist respectively). On the other hand, there was no significant difference in AUC between with AI and without AI for Radiologist 1(0.968 with AI vs. 0.950 without AI; *p* = 0.139).

**Fig.4:**
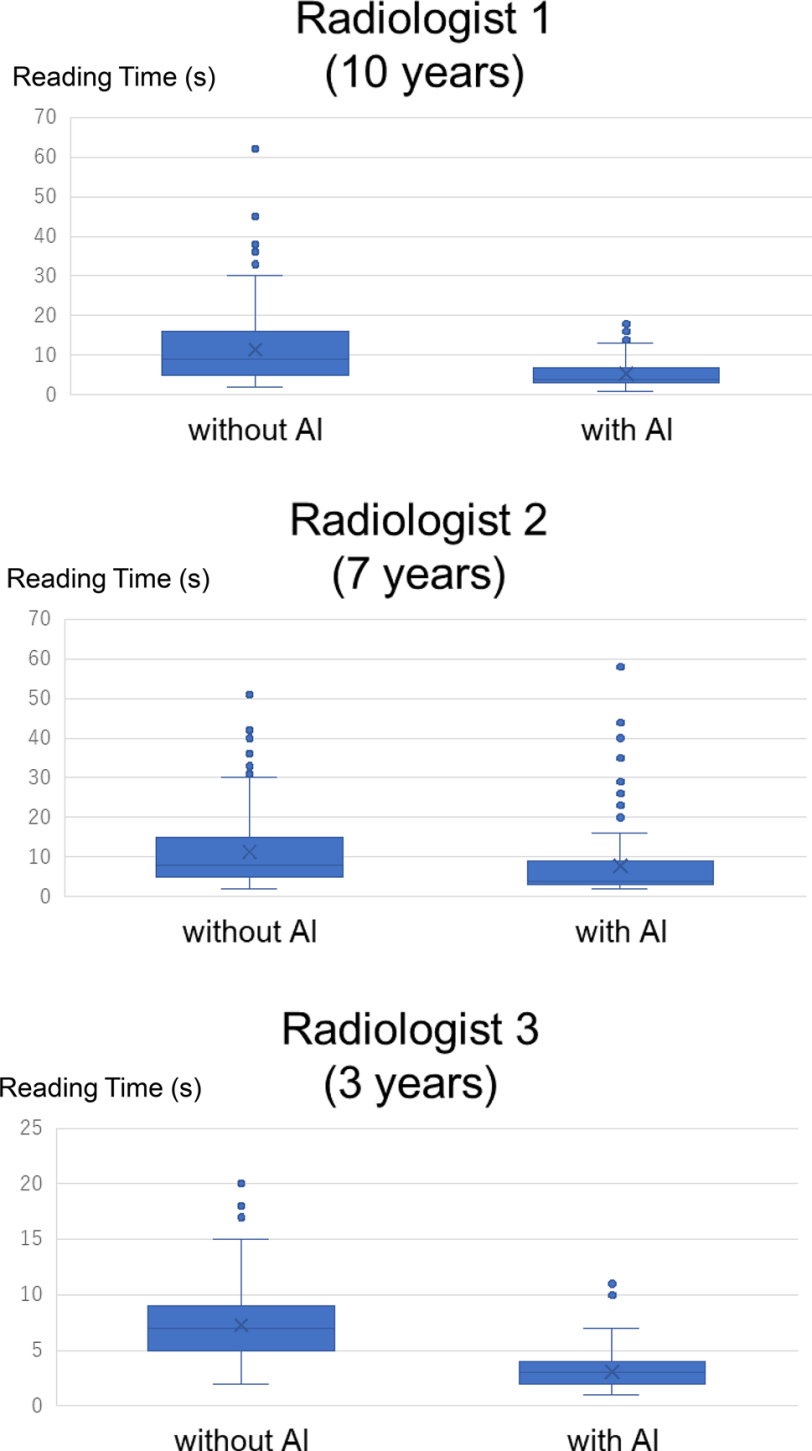
Diagnosis time of the radiologists with or without AI

Figure 4 presents the diagnosis time of the radiologists with or without AI on the same radiologist using box plots. Table 5 summarizes the median diagnosis time. The standalone AI system’s reading time was not aggregated because it was less than 2 s and therefore too short to measure accurately. The AI reduced the diagnosis time among all the radiologists. As previously mentioned, there was no significant difference in diagnostic performance, such as sensitivity and specificity, between AI and Radiologist 1. However, the use of AI substantially reduced the diagnosis time even for Radiologist 1.

**Table 5:** The median diagnosis time with or without AI

Radiologist	without or with AI	Median time (seconds)
Radiologist 1	without AI	9
(10 years)	with AI	4
Radiologist 2	without AI	8
(7 years)	with AI	4
Radiologist 4	without AI	7
(3 years)	with AI	3

*AI* artificial intelligence

## Discussion

The AI system employed in this study demonstrated a high level of accuracy in diagnosing breast masses, comparable to that of expert breast radiologists and superior to that of junior breast radiologists. The AI system supported radiologists’ diagnosis in terms of both accuracy and time, which is also particularly beneficial to younger radiologists. By reducing the time required for each diagnosis, radiologists can increase the number of cases they diagnose per day and allocate more time to cases that require advanced interpretation skills. With the support of the AI system, the readers were able to effectively improve their diagnostic performance, and the AUC for individual readers also improved; however, the improvement for Radiologist 1 did not reach statistical significance. Radiologist 1 has extensive experience in breast ultrasound diagnosis and had the highest AUC among all readers even without the AI system’s support. This suggests that AI assistance may offer less improvement for experienced readers, which aligns with findings mentioned in previous studies on BU-CAD™ [22].

AI applications in breast ultrasound diagnosis have demonstrated various achievements. In the study by Shen et al., an AI system was developed and evaluated using 288,767 breast ultrasound examinations collected at NYU Langone Health in New York, USA [23]. The results showed that the AI system achieved a higher AUC compared to radiologists (0.976 vs. 0.924). Furthermore, with AI assisting radiologists, the false positive rate was reduced by 37.3%, and the number of recommended biopsies was decreased by 27.8%, all while maintaining sensitivity. These findings suggest that AI has the potential to enhance the accuracy of breast ultrasound diagnosis.

Furthermore, AI applications in breast ultrasound extend beyond the detection and diagnosis of breast cancer to include broader fields such as predicting molecular subtypes, assessing chemotherapy response, and predicting lymph node metastasis. AI models designed to identify molecular subtypes, such as triple-negative breast cancer, have demonstrated higher accuracy than conventional ultrasound and, in some cases, surpass the diagnostic capabilities of preoperative core biopsies. Additionally, AI technologies for predicting the efficacy of chemotherapy can evaluate treatment responses in real time and accurately identify non-responders, thereby aiding in the selection of appropriate therapeutic strategies. AI is also being utilized to predict axillary lymph node metastasis, and when combined with ultrasound, it can enhance diagnostic accuracy and potentially reduce unnecessary surgical procedures. The advancement of ultrasound-based AI brings us closer to more accurate and personalized breast cancer care [24].

Several studies have focused on Asian women as their target population. Qiu et al. developed an AI classification model for breast lesions, termed Auto BI-RADS, based on ACR BI-RADS characteristics, and prospectively evaluated its performance [25]. The model was trained using ultrasound dynamic videos of 480 pathologically confirmed breast lesions from 420 patients, equivalent to a total of 18,122 static images. Additionally, a prospective test of Auto BI-RADS was conducted using ultrasound dynamic videos of 292 breast lesions collected from both internal and external hospitals. The results showed that Auto BI-RADS achieved an accuracy of 0.87, a sensitivity of 0.93, and a specificity of 0.81. Moreover, the agreement in BI-RADS category classification between Auto BI-RADS and experienced radiologists (kappa value: 0.82) was higher than the agreement between junior physicians and experienced radiologists (kappa value: 0.60).

In Japan, two AI-based diagnostic support systems for breast imaging have been approved by the Pharmaceuticals and Medical Devices Agency (PMDA) [26, 27]. These two systems are introduced in the following section. One study evaluated a deep learning-based Computer-Aided Detection (CADe) system for breast ultrasound, the BR-FHUS Smart System, using 14,000 positive and 50,000 negative images [26]. The CADe system was trained to detect lesions in real-time using an improved model of YOLOv3-tiny, with significant improvements observed in lesion detection, sensitivity, and specificity when compared to traditional methods. The AUC for image sets with CADe was significantly higher at 0.7726 compared to 0.6304 without CADe, indicating its effectiveness in enhancing diagnostic accuracy (*p* < 0.0001). Another study focused on addressing the issue of inter-observer variability in ultrasound categorization using the BI-RADS [27]. The study developed an AI system capable of distinguishing between BI-RADS 3 or lower and BI-RADS 4a or higher, achieving an AUC of 0.95, with sensitivity and specificity rates of 91.2% and 90.7%, respectively. Notably, the AI system outperformed 20 clinicians in diagnostic accuracy (McNemar test, *p* < 0.001). When comparing the AI system developed in the study by Hayashida et al. [27].with the AI system we used (BU-CAD™), the AUC and sensitivity were approximately equivalent, while the specificity of BU-CAD™ was slightly superior. These findings underscore the potential of AI to improve diagnostic consistency and accuracy in breast imaging, further highlighting the significance of integrating AI systems in clinical practice.

A key point of this study is that the AI support used includes not only CADe (lesion detection) but also CADx (lesion malignancy classification). This system classifies the malignancy of lesion candidates using BI-RADS and provides information on diagnostic characteristics such as lesion margins (e.g., angular, spiculated) and echo patterns (e.g., hyperechoic, hypoechoic, complex cystic and solid).

Previous studies on CADx targeting Japanese patients have focused on evaluating and comparing the diagnostic performance of AI and radiologists. However, this study goes further by investigating whether the use of AI improves diagnostic accuracy and reduces reading time, making it a novel and significant contribution. This approach clarifies the potential benefits of AI-assisted diagnosis in Japanese patients and expands current knowledge in the field.

The findings of this study suggest that AI can enhance the efficiency of radiologists in interpreting ultrasound images, contributing to improved workflow and patient care in breast ultrasound departments. Specifically, AI support is expected to shorten reading time, reduce the need for double reading, and assist in the training of junior radiologists.

There are various predictions about the future of breast cancer care, including the expectation that mammographic screening, which uses radiation, will only be used for a small number of women with low risk and involuted breasts, and that artificial intelligence will play a role not only in predicting individual women’s risk and phenotyping cancer, but also in the acquisition and interpretation of all screening studies [28]. Research on AI for breast ultrasound has been active in recent years [22, 29, 30], but only a few diagnostic support systems have been approved by public institutions. In Japan, there are two AI-based diagnostic support systems for breast imaging approved by PMDA [26, 27]. In the future, AI-based diagnostic support systems are expected to become more commonly used in clinical practice, either by importing existing systems or developing domestic ones.

Our study had several limitations. First, it was a single-facility study with a retrospective design and a relatively small sample size. Additionally, the study was validated using only ultrasound devices from two specific vendors. A larger prospective study involving multiple devices would be more valuable. Furthermore, since the study was conducted in a population with a high proportion of malignant cases, further evaluation is needed to determine whether this AI system is useful in screening populations. The cases in this study were collected from a university hospital (Tokyo Medical and Dental University Hospital), which serves as a specialized diagnostic center. This setting likely contributed to the high proportion of malignant cases observed in the study. Therefore, caution is required when applying these findings to general clinical practice, as the patient population and diagnostic settings may differ significantly. Moreover, the fifth edition of BI-RADS recommends follow-up instead of biopsy for BI-RADS Category 3 lesions with a malignancy probability of less than 2%, to reduce the number of false-positive biopsies. In this study, out of the 92 lesions identified as benign, 77 were diagnosed by follow-up without pathological confirmation, suggesting a slight possibility of false negatives. Lastly, in clinical practice, breast cancer diagnoses are made by considering patient age, medical history, and family history. However, this study focused solely on evaluating the AI system’s support in interpreting breast ultrasound images, without taking such clinical information into account.

## Conclusion

The AI system employed in this study significantly supported radiologists’ diagnoses in terms of both accuracy and diagnosis time, proving particularly beneficial to less experienced radiologists. AI systems have the remarkable potential to revolutionize breast ultrasound diagnostics. With thoughtful attention to ethical, legal, and technical considerations, we can fully realize and maximize their benefits in clinical settings, paving the way for a new era of enhanced diagnostic accuracy and patient care.

## References

[1] Siegel RL, Giaquinto AN, Jemal A. Cancer statistics, 2024. CA Cancer J Clin. 2024;74(1):12–49. 38230766 10.3322/caac.21820

[2] Kornecki A. Current status of breast ultrasound. Can Assoc Radiol J. 2011;62(1):31–40. 20870376 10.1016/j.carj.2010.07.006

[3] Newell MS, Mahoney MC. Ultrasound-guided percutaneous breast biopsy. Tech Vasc Interv Radiol. 2014;17(1):23–31. 24636328 10.1053/j.tvir.2013.12.005

[4] Jones BA, Patterson EA, Calvocoressi L. Mammography screening in African American women: evaluating the research: evaluating the research. Cancer. 2003;97(1 Suppl):258–72. 12491490 10.1002/cncr.11022

[5] Gilbert FJ, Pinker-Domenig K. Diagnosis and staging of breast cancer: When and how to use mammography, tomosynthesis, ultrasound, contrast-enhanced mammography, and magnetic resonance imaging. In: IDKD Springer Series. Cham: Springer International Publishing; 2019. p. 155–66. 32096932

[6] Nara M, Fujioka T, Mori M, Aruga T, Tateishi U. Prediction of breast cancer risk by automated volumetric breast density measurement. Jpn J Radiol. 2023;41(1):54–62. 35913644 10.1007/s11604-022-01320-y

[7] Ohuchi N, Suzuki A, Sobue T, Kawai M, Yamamoto S, Zheng Y-F, et al. Sensitivity and specificity of mammography and adjunctive ultrasonography to screen for breast cancer in the Japan Strategic Anti-cancer Randomized Trial (J-START): a randomised controlled trial. Lancet. 2016;387(10016):341–8. 26547101 10.1016/S0140-6736(15)00774-6

[8] Kubota K. Breast ultrasound in the age of advanced technology and artificial intelligence. J Med Ultrason. 2021;48(2):113–4. 10.1007/s10396-021-01091-5 33907926

[9] Yanagawa M, Ito R, Nozaki T, Fujioka T, Yamada A, Fujita S, et al. New trend in artificial intelligence-based assistive technology for thoracic imaging. Radiol Med. 2023;128(10):1236–49. 37639191 10.1007/s11547-023-01691-w PMC10547663

[10] Yamada A, Kamagata K, Hirata K, Ito R, Nakaura T, Ueda D, et al. Clinical applications of artificial intelligence in liver imaging. Radiol Med. 2023;128(6):655–67. 37165151 10.1007/s11547-023-01638-1

[11] Kitahara H, Nagatani Y, Otani H, Nakayama R, Kida Y, Sonoda A, et al. A novel strategy to develop deep learning for image super-resolution using original ultra-high-resolution computed tomography images of lung as training dataset. Jpn J Radiol. 2022;40(1):38–47. 34318444 10.1007/s11604-021-01184-8 PMC8315896

[12] Tatsugami F, Nakaura T, Yanagawa M, Fujita S, Kamagata K, Ito R, et al. Recent advances in artificial intelligence for cardiac CT: enhancing diagnosis and prognosis prediction. Diagn Interv Imaging. 2023;104(11):521–8. 37407346 10.1016/j.diii.2023.06.011

[13] Oshima S, Fushimi Y, Miyake KK, Nakajima S, Sakata A, Okuchi S, et al. Denoising approach with deep learning-based reconstruction for neuromelanin-sensitive MRI: image quality and diagnostic performance. Jpn J Radiol. 2023;41(11):1216–25. 37256470 10.1007/s11604-023-01452-9 PMC10613599

[14] Yasaka K, Akai H, Sugawara H, Tajima T, Akahane M, Yoshioka N, et al. Impact of deep learning reconstruction on intracranial 1.5 T magnetic resonance angiography. Jpn J Radiol. 2022;40(5):476–83. 34851499 10.1007/s11604-021-01225-2 PMC9068615

[15] O’Connell AM, Bartolotta TV, Orlando A, Jung S-H, Baek J, Parker KJ. Diagnostic performance of an artificial intelligence system in breast ultrasound. J Ultrasound Med. 2022;41(1):97–105. 33665833 10.1002/jum.15684

[16] Barinov L, Jairaj A, Becker M, Seymour S, Lee E, Schram A, et al. Impact of data presentation on physician performance utilizing artificial intelligence-based computer-aided diagnosis and decision support systems. J Digit Imaging. 2019;32(3):408–16. 30324429 10.1007/s10278-018-0132-5 PMC6499739

[17] Mango VL, Sun M, Wynn RT, Ha R. Should we ignore, follow, or biopsy? Impact of artificial intelligence decision support on breast ultrasound lesion assessment. AJR Am J Roentgenol. 2020;214(6):1445–52. 32319794 10.2214/AJR.19.21872 PMC8162774

[18] Uematsu T, Nakashima K, Harada TL, Nasu H, Igarashi T. Comparisons between artificial intelligence computer-aided detection synthesized mammograms and digital mammograms when used alone and in combination with tomosynthesis images in a virtual screening setting. Jpn J Radiol. 2023;41(1):63–70. 36068450 10.1007/s11604-022-01327-5 PMC9813079

[19] Goto M, Sakai K, Toyama Y, Nakai Y, Yamada K. Use of a deep learning algorithm for non-mass enhancement on breast MRI: comparison with radiologists’ interpretations at various levels. Jpn J Radiol. 2023;41(10):1094–103. 37071250 10.1007/s11604-023-01435-w PMC10543141

[20] Ozaki J, Fujioka T, Yamaga E, Hayashi A, Kujiraoka Y, Imokawa T, et al. Deep learning method with a convolutional neural network for image classification of normal and metastatic axillary lymph nodes on breast ultrasonography. Jpn J Radiol. 2022;40(8):814–22. 35284996 10.1007/s11604-022-01261-6

[21] Spak DA, Plaxco JS, Santiago L, Dryden MJ, Dogan BE. BI-RADS® fifth edition: a summary of changes. Diagn Interv Imaging. 2017;98(3):179–90. 28131457 10.1016/j.diii.2017.01.001

[22] Lai Y-C, Chen H-H, Hsu J-F, Hong Y-J, Chiu T-T, Chiou H-J. Evaluation of physician performance using a concurrent-read artificial intelligence system to support breast ultrasound interpretation. Breast. 2022;65:124–35. 35944352 10.1016/j.breast.2022.07.009 PMC9379669

[23] Shen Y, Shamout FE, Oliver JR, Witowski J, Kannan K, Park J, et al. Artificial intelligence system reduces false-positive findings in the interpretation of breast ultrasound exams. Nat Commun. 2021;12(1):5645. 34561440 10.1038/s41467-021-26023-2 PMC8463596

[24] Fruchtman Brot H, Mango VL. Artificial intelligence in breast ultrasound: application in clinical practice. Ultrasonography. 2024;43(1):3–14. 38109894 10.14366/usg.23116 PMC10766882

[25] Qiu S, Zhuang S, Li B, Wang J, Zhuang Z. Prospective assessment of breast lesions AI classification model based on ultrasound dynamic videos and ACR BI-RADS characteristics. Front Oncol. 2023;3(13):1274557. 10.3389/fonc.2023.1274557 PMC10656688 38023255

[26] Fujioka T, Kubota K, Hsu JF, Chang RF, Sawada T, Ide Y, et al. Examining the effectiveness of a deep learning-based computer-aided breast cancer detection system for breast ultrasound. J Med Ultrason. 2023;50(4):511–20. 10.1007/s10396-023-01332-9 PMC10556122 37400724

[27] Hayashida T, Odani E, Kikuchi M, Nagayama A, Seki T, Takahashi M, et al. Establishment of a deep-learning system to diagnose BI-RADS4a or higher using breast ultrasound for clinical application. Cancer Sci. 2022;113(10):3528–34. 35880248 10.1111/cas.15511 PMC9530860

[28] Kuhl CK. What the future holds for the screening, diagnosis, and treatment of breast cancer. Radiology. 2023;306(3): e223338. 36802999 10.1148/radiol.223338

[29] Fujioka T, Mori M, Kubota K, Oyama J, Yamaga E, Yashima Y, et al. The utility of deep learning in breast ultrasonic imaging: a review. Diagnostics (Basel). 2020;10(12):1055. 33291266 10.3390/diagnostics10121055 PMC7762151

[30] Han S, Kang H-K, Jeong J-Y, Park M-H, Kim W, Bang W-C, et al. A deep learning framework for supporting the classification of breast lesions in ultrasound images. Phys Med Biol. 2017;62(19):7714–28. 28753132 10.1088/1361-6560/aa82ec

